# Using infective mosquitoes to challenge monkeys with *Plasmodium knowlesi* in malaria vaccine studies

**DOI:** 10.1186/1475-2875-13-215

**Published:** 2014-06-03

**Authors:** Jittawadee R Murphy, Walter R Weiss, David Fryauff, Megan Dowler, Tatyana Savransky, Cristina Stoyanov, Olga Muratova, Lynn Lambert, Sachy Orr-Gonzalez, Katie Lynn Zeleski, Jessica Hinderer, Michael P Fay, Gyan Joshi, Robert W Gwadz, Thomas L Richie, Eileen Franke Villasante, Jason H Richardson, Patrick E Duffy, Jingyang Chen

**Affiliations:** 1 Walter Reed Army Institute of Research, Silver Spring, MD, USA; 2 Naval Medical Research Center, Silver Spring, MD, USA; 3 NIH, NIAID, The Laboratory of Malaria Immunology and Vaccinology, Rockville, MD, USA; 4 NIH, NIAID, Division of Clinical Research, Biostatistics Research Branch, Bethesda, MD, USA; 5 SAIC-Frederick, Inc, Frederick National Laboratory for Cancer Research, Frederick, MD 21702, USA; 6 NIH, NIAID, The Laboratory of Malaria and Vector Research, Rockville, MD, USA

**Keywords:** Monkey, Rhesus, *Macaca mulatta*, *Plasmodium knowlesi*, *Anopheles dirus*, *Anopheles crascen*s, Vaccine, Methylparaben, Mosquito, Challenge

## Abstract

**Background:**

When rhesus monkeys (*Macaca mulatta)* are used to test malaria vaccines, animals are often challenged by the intravenous injection of sporozoites. However, natural exposure to malaria comes via mosquito bite, and antibodies can neutralize sporozoites as they traverse the skin. Thus, intravenous injection may not fairly assess humoral immunity from anti-sporozoite malaria vaccines. To better assess malaria vaccines in rhesus, a method to challenge large numbers of monkeys by mosquito bite was developed.

**Methods:**

Several species and strains of mosquitoes were tested for their ability to produce *Plasmodium knowlesi* sporozoites. Donor monkey parasitaemia effects on oocyst and sporozoite numbers and mosquito mortality were documented. Methylparaben added to mosquito feed was tested to improve mosquito survival. To determine the number of bites needed to infect a monkey, animals were exposed to various numbers of *P. knowlesi*-infected mosquitoes. Finally, *P. knowlesi*-infected mosquitoes were used to challenge 17 monkeys in a malaria vaccine trial, and the effect of number of infectious bites on monkey parasitaemia was documented.

**Results:**

*Anopheles dirus, Anopheles crascens*, and *Anopheles dirus X* (a cross between the two species) produced large numbers of *P. knowlesi* sporozoites. Mosquito survival to day 14, when sporozoites fill the salivary glands, averaged only 32% when donor monkeys had a parasitaemia above 2%. However, when donor monkey parasitaemia was below 2%, mosquitoes survived twice as well and contained ample sporozoites in their salivary glands. Adding methylparaben to sugar solutions did not improve survival of infected mosquitoes. *Plasmodium knowlesi* was very infectious, with all monkeys developing blood stage infections if one or more infected mosquitoes successfully fed. There was also a dose-response, with monkeys that received higher numbers of infected mosquito bites developing malaria sooner.

**Conclusions:**

*Anopheles dirus, An. crascens* and a cross between these two species all were excellent vectors for *P. knowlesi*. High donor monkey parasitaemia was associated with poor mosquito survival. A single infected mosquito bite is likely sufficient to infect a monkey with *P. knowlesi*. It is possible to efficiently challenge large groups of monkeys by mosquito bite, which will be useful for *P. knowlesi* vaccine studies*.*

## Background

In 1931, *Plasmodium knowlesi* was identified as an infection of monkeys [1], and the following year it was experimentally transmitted to humans [2]. The first naturally occurring case of *P. knowlesi* in humans was reported in 1965 [3]. In 2004, it was discovered that *P. knowlesi* frequently infects humans in Southeast Asia [4], a finding which required PCR to distinguish *P. knowlesi* from *Plasmodium malariae*.

*Plasmodium knowlesi* infections in rhesus monkeys have been used as a model system to test malaria vaccines [5-24]. The advantages of this model system are that the immune systems of rhesus monkeys and humans are similar, and that *P. knowlesi* sporozoites are extremely infectious. Often, monkeys are challenged with malaria by the intravenous (iv) inoculation of *P. knowlesi* sporozoites dissected from infected mosquitoes. Intravenous delivery of sporozoites to monkeys is quick, and 40 monkeys can be injected in two hours. Intravenous delivery also allows control of sporozoite doses. In vaccine trials at our laboratory challenge with 100 *P. knowlesi* sporozoites has been routine. With this dose, 56/56 control monkeys in 12 separate experiments (10 published [5-9] and two unpublished) have all developed blood stage infections (estimated probability of infection 100%, 95% CI 93.6%, 100%). In one of these trials, 80% of vaccinated monkeys were protected against malaria, showing that this dose of sporozoites is not large enough to overwhelm the immune response. Because of the high infectivity, only several thousand sporozoites are needed to challenge the 30-40 monkeys in a vaccine trial. As *P. knowlesi* produces many thousands of sporozoites per mosquito, few mosquitoes are required to produce the sporozoites needed on the day of challenge.

However, the authors are now convinced that for testing of some malaria vaccines, challenge of monkeys should be by mosquito bite. Vanderberg *et al.*[25-29] and others [30-34] have shown how malaria sporozoites are deposited into the skin, blood, and other tissues during probing. They have also shown that antibodies can trap sporozoites in the skin, preventing them from reaching the liver. Intravenous injection of sporozoites bypasses the barrier of the skin, and might underestimate the effect of vaccines based on antibodies that neutralize sporozoites [28]. Not all malaria vaccines produce antibodies to sporozoites. Some vaccines, such as DNA plasmid vaccines, produce few or no antibodies in monkeys or humans, and seem to induce only T cell immune responses. Other vaccines contain antigens expressed in malaria blood or liver stages but not in the sporozoite. Intravenous challenge may be a reasonable method for testing such vaccines. However, many malaria sporozoites inoculated by mosquito bite end up in the skin or draining lymph nodes and not in the blood stream [31,35]. It is theoretically possible that the immune responses to these lymphatic sporozoites might increase or decrease vaccine responses against malaria antigens of any parasite stage. *Plasmodium knowlesi* liver stages last only 4.5 days [1], so it is unlikely that vaccine responses would be altered by lymphatic sporozoites in time to affect intra-hepatic immune responses. In contrast, *P. knowlesi* blood stages can persist for weeks or months, and immune responses to lymphatic sporozoites could conceivably alter blood stage immunity and have consequences for vaccine efficacy.

The goal of these studies was to develop a *P. knowlesi* challenge model for rhesus monkeys using the bite of infected mosquitoes. While monkeys have been infected with *P. knowlesi* by mosquito bite for biologic studies, mosquito bite challenge has never been used for testing *P. knowlesi* malaria vaccines. Theoretically, the malaria exposure of monkeys in a vaccine trial should be adequate to show a statistically significant difference in infection between the control and vaccinated group. If the rate of infection is too low, very large group sizes are required to detect differences. If malaria exposure is too high, it may overwhelm the vaccine or not reflect actual transmission in endemic settings. In practice, with the small group sizes of five to eight monkeys in malaria studies, the goal is to use a malaria exposure which will infect all control monkeys with high probability.

As mentioned above, there is high confidence that 100 *P. knowlesi* sporozoites given iv will infect a rhesus monkey. It is estimated that 1-2.5 sporozoites per second are injected from the mosquito during secretion of saliva while probing the skin [27], which may be a physical limitation based on sporozoite diameter and mosquito anatomy. Thus, in the minutes required for one mosquito to probe the skin, several hundred sporozoites might leave the mosquito, although not all of these would reach the bloodstream. Thus, one mosquito bite should be adequate to reliably cause a blood stage infection with *P. knowlesi.*

In 1968, Chin *et al.*[36] exposed five human volunteers to *P. knowlesi*-infected *Anopheles balabacencis balabacencis* (= *Anopheles dirus*) mosquitoes. The volunteers received from one to nine infective bites. All five volunteers developed *P. knowlesi* infections in the blood. Thus it appeared possible that a single bite by a *P. knowlesi*-infected mosquito might be adequate to infect a monkey. The goal of the present study was determine the exposure to mosquitoes carrying *P. knowlesi* necessary to infect naive monkeys with high probability.

*Anopheles dirus* mosquitoes require mating by hand when grown in the laboratory, which is time-consuming. This led us to try other mosquito species and strains which are easier to rear. *Anopheles crascens* and a cross between *An. dirus* and *An. crascens* (termed *Anopheles dirus* X) were excellent hosts for *P. knowlesi*, and could be reared without forced mating. Unfortunately, *P. knowlesi* infections caused high mortality in mosquitoes. It was found that the parasitaemia level of the donor monkey affected the survival of infected mosquitoes and a low donor parasitaemia produced adequate numbers of sporozoites without great mosquito mortality. Methylparaben (MPB) added to sugar solutions [37] did not consistently reduce mortality. Finally, *P. knowlesi*-infected mosquitoes were used to challenge 17 monkeys in a vaccine study, and it was seen that increased numbers of bites led to more rapid onset of parasitaemia.

## Methods

### Monkeys

The experiments described in this paper were carried out at two facilities, the Walter Reed Army Institute of Research/Naval Medical Research Center, Silver Spring Maryland, USA (WRAIR/NMRC), or the Laboratory of Malaria Immunology and Vaccinology (LMIV) and the Laboratory of Malaria and Vector Research (LMVR), NIAID, NIH, Rockville, Maryland, USA. Adult *Macaca mulatta* bred in the US from Indian stock and maintained in facilities accredited by the Association for Assessment and Accreditation of Laboratory Animal Care were used in all studies. The experiments were conducted in compliance with the Animal Welfare Act and in accordance with the principles set forth in the “Guide for the Care and Use of Laboratory Animals,” Institute of Laboratory Animals Resources, National Research Council, National Academy Press, 1996. Experiments were approved by the Institutional Animal Care and Use Committee, WRAIR/NMRC or the NIAID Institutional Animal Care and Use Committee, NIH, depending on the location of the experiments. *M. mulatta* develops higher parasitaemias than do *Macaca fascicularis* and *Macaca nemestrina,* which are the natural hosts of *P. knowlesi*. All animals with malaria infections were closely monitored and treated with anti-malarial drugs, and adjunctive therapy was given as needed.

### Malaria parasites

*Plasmodium knowlesi* H strain parasites were used in these experiments, and were derived from stocks at NIH. Oocysts were counted by removing the midgut of mosquitoes on day 7 after infection and counting under a dissecting microscope. Sporozoites in salivary glands were counted by removing the glands on day 14 and estimating the numbers of sporozoites using the following scale: 0 for none, 1 for 1-10, 2 for 11-100, 3 for 101-1,000, 4 for 1,001-10,000, 5 for >10,000.

### Monitoring and treatment of malaria infections

After sporozoite challenge, from day 6 to day 30 blood was obtained by skin prick for thin film malaria slides. After Giemsa-staining, blood was examined under x1,000 magnification until an estimated 20,000 red cells on thin film were examined. Infected animals were treated with artesunate (5 mg/kg single dose) and chloroquine base (45 mg/kg total dose divided over five days). Animals were monitored for 30 days to ensure effectiveness of treatment in clearing malaria infections.

### Mosquitoes

*Anopheles dirus* (= *An. dirus* A = *An. balabascensis balabascensis* ) were provided by the Malaria Research and Reference Reagent Resource Center**. ***Anopheles crascens* and *An. dirus* X (‘cross’) were provided by LMVR, NIH. *Anopheles dirus* X were produced at NIH by a mating of *An. dirus* males and *An. crascens* females. *Anopheles stephensi* and *Anopheles gambiae* were provided by WRAIR/NMRC.

### Production of *Plasmodium knowlesi* infected mosquitoes

All mosquitoes were infected with *P. knowlesi* malaria 4-6 days after adults emerged from pupae. Prior to infection, mosquitoes were fed 10% sucrose at WRAIR/NMRC, or 10% sucrose or 10% Karo brand syrup at NIH. Previously splenectomized monkeys were infected by iv injection of cryopreserved red blood cells infected with *P. knowlesi*. Splenectomy allows repeated infections with *P. knowlesi* without the animals developing immunity. In the authors’ experience, splenectomy does not affect gametocyte numbers or infectivity to mosquitoes. Feeding of mosquitoes on *P. knowlesi*-infected monkeys was carried out at 10 pm. 30-160 mosquitoes in a pint carton were starved for eight hours prior to the feed. Feeding was on a monkey anesthetized with ketamine and acepromazine. Hair was clipped on the chest or abdomen, and cups pressed against the skin under drapes for darkness. After feeding for 15-30 minutes, mosquitoes not engorged with blood were removed, and the remaining mosquitoes were maintained at 26°C and 85% humidity. Cotton pads soaked in sugar solution were changed daily. In some experiments, 10% sucrose was supplemented with methylparaben (MPB) beginning on the day after feeding. The MPB solution was made by adding 1gm of MPB (Sigma-Aldrich Corps. Louis, MO, USA) to 500 ml of a 10% glucose solution, filter sterilizing, and storing at 4°C.

### Infection of monkeys in the vaccine study by mosquito bite challenge

*Anopheles dirus* X mosquitoes were used on days 14-16 after they had fed on a *P. knowlesi*-infected monkey. Mosquitoes were transferred to pint cups, starved of food and water overnight, and allowed to feed at 8 am on the chest or abdominal skin of monkeys. Monkeys were anesthetized with ketamine and acepromazine. After feeding, mosquitoes were dissected to determine the presence of *P. knowlesi* sporozoites in the salivary glands, and whether blood was visible in the midgut, as a measure of successful feeding. Monkeys were followed by blood film for 30 days to determine whether they became infected with malaria. In the preliminary studies, containers with three to 20 mosquitoes were exposed to each monkey for 15-30 minutes. In the vaccine challenge study, containers with five mosquitoes were exposed to each monkey for 15 min, in two groups of 8 and 9 monkeys.

### Statistics

**Figure 1:**
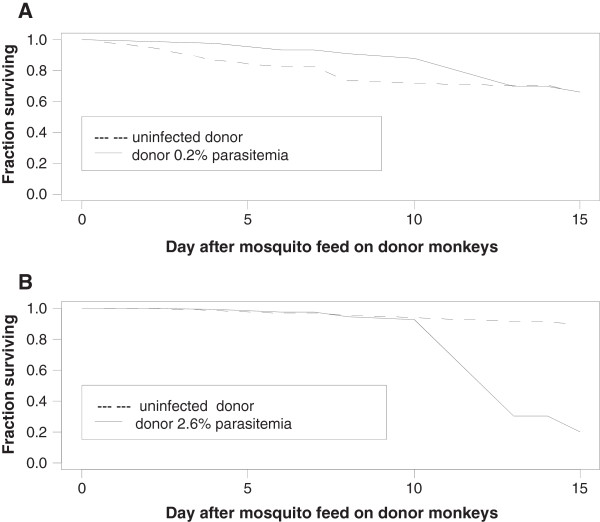
**Survival of heavily and lightly infected mosquitoes.***Anopheles dirus* mosquitoes were put into several pint containers and allowed to feed on a *P. knowlesi* infected monkey on consecutive nights, and survival to day 15 was monitored. For each group between 140 and 160 mosquitoes were fed. Figure 1**A** shows the fraction of mosquitoes surviving from the first night feeds, either on an uninfected monkey or a *P. knowlesi*-infected monkey (parasitaemia of 0.2%). Figure 1**B** shows survival of mosquitoes fed on the same two monkeys one day later, when the parasitaemia in the *P. knowlesi*-infected monkey was 2.6%. Because there was a strong day effect (logrank tests showed significant differences between control groups by day), we used a Cox frailty model using only parasitaemia level of the donor monkey, and with a separate frailty parameter for the four groups. There was a significant difference between the 2.6% group and the combined controls (p = 0.038), but no significant difference between the 2.6% group and the 0.2% group (p = 0.18).

Exact binomial confidence intervals were used to calculate the probability of infection after iv sporozoite injection. For Figure 1, non-parametric maximum likelihood estimators (NPMLEs) of survival were used (i.e., Kaplan-Meier for grouped right-censored data). Because there is a high correlation of the survival times of mosquitoes fed on the same monkey on the same day, standard survival models could not be used. For survival analyses on mosquitoes, Cox frailty models were used, with frailty effects for mosquitoes fed on the same day on the same donor monkey. In Table 1, Mann-Whitney U test tested for differences between continuous variables. In Table 2, the NPMLE for the proportion surviving to day 14 for each cup was calculated first. Then the mean of those proportions was taken within each mosquito species and feed group. The P-value is from the linear model with those proportions as response, controlling for mosquito species. A Cox frailty model with a random effect for mosquito group (a monkey donor on a specific day) was used to estimate the independent effects of species, experiment and donor parasitaemia on mosquito survival to day 15 after infection. A Cox regression analysis was used to calculate the effect of number of infected mosquito bites on time to first parasite in the blood. Calculations were done in R version 3.0.1 (using the survival and interval packages) or SAS v. 9.3.

**Table 1:** ***Plasmodium knowlesi *** **infections of mosquitoes fed on donor monkey on consecutive nights from Figure**1

	**1**^**st **^**night feed**	**2**^**nd **^**night feed**	
Monkey donor parasitaemia	0.2%	2.6%	
Median number of Oocysts (interquartile range)	6.5 (2, 9.75)	80(72.5, 100)	p <0.0001
Sporozoites median grade (interquartile range)	1.5 (1,2)	3 (2.25,3.75)	p = 0.018

The results of dissections from the mosquitoes groups shown in Figure 1A and B. The oocyst counts are from 10 mosquitoes on day 7, and the sporozoite grading in the salivary glands from 10 mosquitoes on day 14. The mosquitoes fed on the high parasitaemia day produced more oocysts (P < 0.0001, Mann-Whitney U test), and sporozoites (P = 0.018, Mann-Whitney U test).

**Table 2:** The effect on survival of adding methyparaben to mosquito feed

	**Exp 1a**	**Exp 1b**	**Exp 2**	**Exp 3a**	**Exp 3b**	**Exp 3c**	**Exp 3d**	**Exp 3e**	**Exp 3f**
Mosquito species		dirus X	dirus X	crascens	dirus	dirus X	dirus	dirus	dirus X
Mosquito age at feed	5 days	8 days	5 days	5 days	5 days	5 days	5 days	5 days	5 days
Monkey donor parasitaemia	12%	12%	1.30%	5.50%	5.50%	5.50%	0.30%	0% (control)	0% (control)
% surviving fed sugar only (# replicate cups)	53% (4)	88% (4)	87% (4)	36% (1)	41% (2)	22% (1)	64% (1)	88% (1)	24% (1)
% surviving fed sugar + MPB (# replicate cups)	91% (6)	45% (2)	81% (14)	20% (1)	29% (2)	0% (1)	41% (1)	49% (1)	2% (1)
	p < 0.001	p = 0.04	p = 0.15	p = .03			NA	p = .18	

Methylparaben (MPB) was added to the sugar solution used for feeding mosquitoes starting the day they were infected with *P. knowlesi.* Mosquitoes from the same batch were maintained on sugar solution without MPB. Experiments 1, 2 and 3 were on different dates, and have been subdivided by mosquito species and donor monkey. In some experiments, there were several replicate cups of mosquitoes for each feeding group. Sugar solutions on cages were changed daily. Percentage surviving is the mean from each cup to day 14. P-values were calculated as described in Methods section. For experiment 3d, there were only 2 cups and no statistical test could be done. MPB only improved survival in one of nine sub-experiments.

## Results

### Choice of mosquito species to infect with *P. knowlesi*

The first goal was to find a mosquito vector for *P. knowlesi* that was easy to maintain. *Anopheles dirus* is an excellent vector for *P. knowlesi*, but it requires hand mating in the laboratory, which limits its usefulness. Efforts were focused on mosquitoes that were easy to rear. In the first studies, an attempt was made to infect *An. stephensi and An. gambiae,* which had been reported to support *P. knowlesi* sporozoite development [38-41] and which were readily available. Although these mosquitoes produced large numbers of oocysts, only an occasional sporozoite was found in the salivary glands, and work was not continued with these species. Three types of mosquitoes produced large numbers of *P. knowlesi* sporozoites: *An. dirus*, *An. crascens*, and a cross-mating of *An. dirus* males and *An. crascens* females termed *An. dirus* X (‘cross’). Both *An. crascens* and *An. dirus X* mosquitoes mate well in the laboratory and were more easily produced in large numbers than *An. dirus*.

Survival of *P. knowlesi*-infected mosquitoes of *An. dirus*, *An. dirus* X, and *An. crascens* were compared in five experiments at high and low donor parasitaemia. In monkeys, *P. knowlesi* parasitaemia increases 10-fold every day, so groups of mosquitoes fed on the same monkey on consecutive nights provided the low and high parasite groups. In some experiments mosquitoes were fed on an uninfected monkey as a control. Figure 1 and Table 1 show representative data from one of the experiments.

Mosquitoes feeding on donor monkeys with a high parasitaemia had large numbers of oocysts in the midgut dissections on day 7, high levels of sporozoites on gland dissections on day 14, but poor survival. Mosquitoes fed on the same donor monkeys a day earlier when the parasitaemia was lower, had smaller numbers of oocysts and sporozoites, and better survival. This was true for *An. dirus*, *An. dirus* X, and *An. crascens* mosquitoes. Overall, 16 groups of mosquitoes were fed on monkeys with a parasitaemia greater than 2%, and the median of the day 14 survival was 32% (interquartile range [IQR]: 23%, 38%). All three types of mosquitoes produced large numbers of *P. knowlesi* sporozoites (see Additional file 1 for complete data). Survival data from the five experiments were analysed using a Cox Frailty model. Higher donor parasitaemia was significantly associated with lower mosquito survival (p-value <0.001) after correcting for mosquito species effects and experiment effects.

### Methylparaben as an additive to improve mosquito survival

Methylparaben (MPB) is an antibacterial and antifungal agent which can improve the survival of malaria-infected mosquitoes when maintenance sugar solutions are changed infrequently [37]. The addition of MPB to the sugar solution was tested in our laboratories where sugar pads are changed daily. Three experiments were done adding MPB at 2 gm/L to the sugar solutions of some mosquitoes while others received standard sugar solution. Table 2 shows the effect of MPB on the percentage of mosquitoes surviving to day 14 after *P. knowlesi* infection. In Experiment 1a using mosquitoes aged 5 days at feed, MBP enhanced survival. In Experiment 1b using mosquitoes aged 8 days at feed there was a significant worsening of survival (p = 0.04). In Experiment 2, there was no significant difference in survival. Experiment 3 had six subgroups of mosquitoes fed on monkeys with 5.5%, 0.3% or no malaria infection. In all the subgroups of Experiment 3, MPB decreased the fraction of mosquitoes surviving until day 14, but the difference was significant only in the mosquitoes fed on the donor monkey with 5.5% parasitaemia. Overall, the effects of MPB on survival were inconsistent.

### How many bites from *P. knowlesi*-infected mosquitoes are required to transmit malaria to a monkey?

Over several years, 49 monkeys were exposed to varying numbers of mosquitoes (3 to 20) for 15 to 30 min. Immediately after, mosquitoes were dissected to determine the numbers of sporozoites in the salivary glands and the presence of ingested blood as a mark of successful feeding. Monkeys were followed for 30 days to determine if parasitaemia developed. The results of mosquito dissections and monkey infections are summarized in Table 3 (see Additional file 2 for complete data). Exposures were placed into Categories A, B, C or D of decreasing intensity. Category A exposures were the most intense, with two or more dissected mosquitoes having both *P. knowlesi* sporozoites and ingested blood as a sign they had probed and injected sporozoites. Nine exposures were in Category A, and all led to *P. knowlesi* infections in the monkeys. Category B exposures had only one mosquito with both sporozoites and ingested blood. Eight exposures were in Category B and all eight led to monkey infections. Category C exposures were those where one or more mosquitoes carried *P. knowlesi* sporozoites, even though it had not ingested blood. Ten exposures were in Category C, and 4 of these led to infected monkeys. Finally, Category D exposures were those where upon dissection no mosquitoes were found to carry *P. knowlesi* sporozoites. 22 exposures were in Category D, and 1 of these monkeys developed a *P. knowlesi* infection.

**Table 3:** **Summary of exposure to *P. knowlesi*-infected mosquitoes vs. monkey infection**

**Exposure category**	**# Mosquitoes (+sporozoites/+blood)**	**# Mosquitoes (+sporozoites/no blood)**	**# Monkey exposures**	**% Monkeys infected**
A	≥ 2	0 to 10	9	100
B	1	0 to 8	8	100
C	0	≥ 1	10	40
D	0	0	22	4

From 3-20 mosquitoes infected with *P. knowlesi* were exposed to a monkey for 15-30 minutes on 49 occasions. Mosquitoes were dissected to grade the sporozoites in the salivary glands, and identify ingested blood in the mosquito midgut. Monkeys were followed for 30 days to see if they developed parasites in the blood. Category A exposures had two or more mosquitoes containing both sporozoites and blood. Category B exposures had only one mosquito with both sporozoites and blood. Category C exposures had some mosquitoes with sporozoites but none of these had ingested blood. Category D exposures had no mosquitoes with any sporozoites in the salivary glands, nor any mosquitoes with ingested blood.

Category B exposures show that even a single *P. knowlesi*-infected mosquito feeding is enough to transmit malaria to a non-immune monkey. Of the eight mosquitoes positive for both sporozoites and ingested blood in Category B, five had only grade 2 sporozoites (11-100) in the salivary gland dissections (the remaining three mosquitoes had *P. knowlesi* sporozoites but were not quantitated). The results from Category C exposures were even more surprising. Mosquitoes inject sporozoites while they are probing the skin looking for a blood vessel. Monkey infections from Category C exposures occurred without mosquitoes having ingested blood, so presumably the sporozoites which initiated these infections were transmitted to the monkey during probing which did not result in blood feeding. That 40% of these exposures led to monkey infections shows that successful feeding is not required for transmission of *P. knowlesi*. In theory, Category D exposures should not have resulted in any monkey infections, but one of 22 monkeys became infected. It is probable that the mosquito dissections missed a very small number of sporozoites or that all sporozoites were injected into the monkey during feeding. Overall, we conclude that *P. knowlesi* can be transmitted to monkeys extremely efficiently by *P. knowlesi-*infected mosquitoes. Feeding by a single infected mosquito carrying only modest numbers of sporozoites consistently transmitted malaria to monkeys.

### Feeding rates of mosquitoes at day 0 and day 14

To produce *P. knowlesi* sporozoites, mosquitoes were used 4-6 days after they emerged from pupae to adult. On the day of feeding, it was usual to have 95% of these young mosquitoes take a blood meal. Unfed mosquitoes were then removed and the mosquitoes kept for 14 days while oocysts and then sporozoites developed. Mosquitoes were used to infect monkeys by bite on day 14-16. Blood feeding rates for these older *P. knowlesi*-infected mosquitoes was near 50% but ranged from 10 to 90%.

### Using *P. knowlesi*-infected mosquitoes in a malaria vaccine challenge in monkeys

Based on experience outlined in Table 3, a mosquito bite challenge was planned to test a monkey malaria vaccine based on the *P. knowlesi* sporozoite circumsporozoite protein. Details of the study are being published elsewhere. In this paper, technical aspects of the malaria challenge by exposure to *P. knowlesi*-infected mosquitoes will be discussed.

Seventeen monkeys were challenged with malaria by mosquito exposure. Six monkeys had received a control vaccine and 11 had received malaria vaccine. It was decided to aim for an exposure that would result in blood ingestion by at least two mosquitoes carrying *P. knowlesi* sporozoites. Each monkey was exposed to five *An. dirus* mosquitoes for 15 minutes as described in the methods section. Results are shown in Table 4.

**Table 4:** Results of a mosquito bite challenge in 17 rhesus monkeys

**Monkey ID**	**Vaccine**	**# Mosquitoes with both sporozoites and ingested blood**	**Sporozoite grade of blood feeding mosquitoes**	**Day 1**^**st **^**parasite seen in blood**
1	Control	2	4,4	9
2	Control	3	4,4,4	8
3	Control	3	4,4,4	8
4	Control	4	4,4,4,4	8
5	Control	4	4,4,4,4	7
6	Control	4	2,4,4,4	9
7	Vaccine	2	4,4	7
8	Vaccine	3	4,4,4	7
9	Vaccine	3	4,4,4	9
10	Vaccine	3	2,4,4	8
11	Vaccine	4	2,3,4,4	8
12	Vaccine	4	4,4,4,4	9
13	Vaccine	4	4,4,4,4	7
14	Vaccine	4	3,4,4,4	7
15	Vaccine	5	4,4,4,4,4	9
16	Vaccine	5	2,4,4,4,4	7
17	Vaccine	5	4,4,4,4,4	7

17 rhesus monkeys were exposed to five *P. knowlesi* infected mosquitoes for 15 minutes, in two groups of 8 and 9 monkeys each. The six control monkeys and 11 monkeys receiving malaria vaccine were challenged in random order, but are shown by group for ease of analysis. Mosquitoes were dissected to determine sporozoite grade in the salivary glands and the presence of ingested blood. All 17 monkeys developed parasites in the blood, and the first day parasites were detected is recorded.

The 17 monkeys were challenged in random order but are listed in Table 4 by vaccine group for clarity. After feeding, when the mosquitoes were dissected, from 2 to 5 carried both sporozoites and ingested blood in containers from each of the 17 monkeys. Of the 85 mosquitoes in the challenge, 62 were found to have ingested blood for a bite rate of 73%. All monkeys developed *P. knowlesi* malaria parasites in the blood on day 7 to 9 after exposure. There was no significant difference in time to first parasitaemia in control *versus* vaccinated monkeys (Logrank test, p = 0.46).

### Does feeding by larger numbers of infected mosquitoes lead to more rapid onset of parasitaemia?

When sporozoites are injected by mosquito, they travel quickly to the liver, and begin to develop inside hepatocytes. Hepatocyte development averages 4.5 days for *P. knowlesi*[1], after which thousands of merozoites from each hepatocyte enter the blood and infect red blood cells. In theory, monkeys with few infected hepatocytes will have lower numbers of merozoites entering the blood than monkeys with large numbers of infected hepatocytes. Since a constant number of red blood cells on each slide is examined, the injection of larger numbers of sporozoites would be expected to lead to earlier detection of parasites.

**Figure 2:**
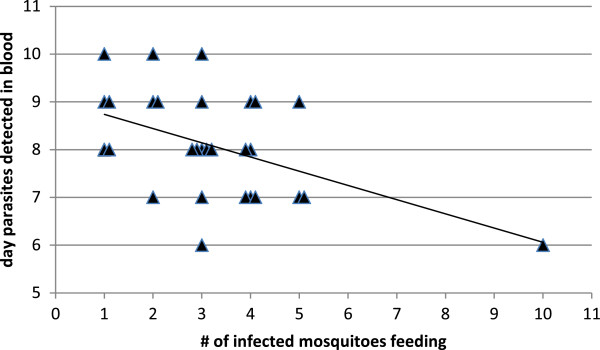
**Effect of number of infected mosquitoes feeding on day parasites detected in blood.** Thirty monkeys were infected with *P. knowlesi* by the bite of from 1 to 10 mosquitoes which had sporozoites in the salivary glands and had ingested blood. The day on which the first *P. knowlesi* parasites were found in the blood is plotted against the number of infectious bites. Increasing numbers of bites lead to earlier detection of parasites in the blood. Cox regression analysis shows the number of infected mosquito bites increased the hazard of parasites appearing in the blood (p = 0.01).

The number of infected mosquito bites correlated with the time when parasites were first detected in the blood. Of the 49 infections of non-immune monkeys, 13 had complete data on both number of infected mosquito bites and day of first parasitaemia. Combining these 13 with the 17 monkeys from vaccine challenge, and using Cox regression, effects of experiment and number of mosquito bites on time to first detection were examined. As there was no significant effect of experiment, a simpler Cox model was used with only number of mosquito bites as a predictor. Figure 2 shows a graph of these data. Each additional bite increased the hazard by a factor of 1.41 (95% CI: 1.08, 1.82). As it was possible that the monkey receiving 10 mosquito bites was driving the results, the analysis was repeated without this data point but the results were similar (HR = 1.28, 95% CI: 0.95, 1.73). The authors conclude that increasing numbers of infected mosquito bites lead to earlier detection of *P. knowlesi* in the blood.

## Discussion

### Survival of *P. knowlesi*-infected mosquitoes

The aim of these studies was to learn how to challenge monkeys with the bite of *P. knowlesi-*infected mosquitoes for vaccine studies. However, the first problem was to produce enough *P. knowlesi*-infected mosquitoes that survived until sporozoites appeared in the salivary glands. The high death rate of *P. knowlesi*-infected mosquitoes was something of a surprise. In the 1960’s and 1970’s, *An. dirus sensu lato* produced large numbers of *P. knowlesi* sporozoites without excessive losses of infected mosquitoes (Robert Gwadz, personal communication). These mosquitoes often carried several hundred oocysts, and dissections of salivary glands yielded up to 250,000 *P. knowlesi* sporozoites per mosquito. Why are the current death rates of the infected mosquitoes so high? It is possible that the current *P. knowlesi* and mosquito strains available are different from those used 40 years ago. However, this history gave hope that we could produce the numbers of *P. knowlesi*-infected mosquitoes required for vaccine studies.

Mosquitoes fed on monkeys with a high parasitaemia developed large numbers of oocysts, but had high death rates during the second week, so that by 14 days after the feed there were sometimes only 10% of mosquitoes surviving. Control groups which were fed on uninfected monkeys or on monkeys with a lower parasitaemia typically had survival of greater than 70%. This difference in mortality between heavily and lightly infected mosquitoes happened in all the species and strains of mosquitoes we tested. It also occurred in both insectaries used for these studies, the one at WRAIR/NMRC in Silver Spring MD, and the other at NIH in Rockville MD. It is not understood what is causing the death of heavily infected mosquitoes. One possibility is that there is something toxic in the blood of monkeys that have high levels of *P. knowlesi* infection, such as a cytokine [42], which might be detrimental to mosquitoes. However, if a toxic substance was ingested, mosquito mortality might be expected to be highest soon after they fed on an infected monkey. However, the excess mosquito deaths happened after the first week of oocyst development, so it is not likely that a toxin from the monkeys is responsible for killing mosquitoes.

The large numbers of *P. knowlesi* oocysts may be contributing to mosquito death, but it seems less likely that the large numbers of sporozoites are harmful to the mosquitoes. Mortality in *P. knowlesi*-infected mosquitoes typically is not high during the first week, but increases 8-12 days after the feed on the infected monkey. During these days, oocysts are large in size but sporozoites have just begun to emerge into the haemolymph and enter the salivary glands. Mortality and stress from oocysts is supported by the results of the one experiment that included *An. stephensi. Plasmodium knowlesi* infection produced large numbers of oocysts in *An. stephensi*, but no sporozoites in the salivary glands. However, *An. stephensi* mosquitos fed on a control uninfected monkey had a survival rate to day 15 of 41.6% while mosquitos feeding on a monkey with 2.6% *P. knowlesi* infection had only a 15.4% survival rate (p < 0.001, Cox frailty model). Although this is data from a single experiment, it supports the theory that the excess mortality to is due to oocysts, not sporozoites.

This is not the first example of intense malaria infections increasing mortality in *An. dirus* mosquitoes. In 1986, Klein *et al.*[43] reported that when *An. dirus* were infected with *Plasmodium cynomolgi*, there was excess mortality in the infected groups. This mortality was only seen in mosquito groups with greater than 10 oocysts. Survival rates in all groups were similar during the first week, and only increased in the heavily infected mosquitoes beginning after the first 8 days of incubation. These results with *P. cynomolgi* infection exactly parallel the results with *P. knowlesi* infections of mosquitoes.

Other studies have looked at the role of malaria infection in mosquito survival. In a 2002 meta-analysis, Ferguson and Read [44] showed that although half of the studies reported no effect of *Plasmodium* on survival, the other half reported shorter lifespans in infected mosquitoes. The negative effects of *Plasmodium* on survival were more likely to appear in non-natural mosquito–parasite combinations, but the authors were unsure of the cause of the discrepant results.

Both *An. dirus* and *An. crascens* have been found infected with *P. knowlesi* in the wild [45,46]. While they may not always be the primary vectors carrying *P. knowlesi*, it seems unlikely that the high mortality seen in this study is due to an artificial pairing of mosquito and parasite.

Two recent studies have looked at mortality in malaria-infected mosquitoes subjected to additional stress. Aboagye-Antwi *et al.*[47] caught *An. gambiae* in Mali and studied survival of mosquitoes with or without naturally occurring *P. falciparum* infections. They found that when water was provided, there was little difference in mortality between mosquitoes carrying malaria oocysts and uninfected mosquitoes. However, when water was withheld, the mosquitoes with oocysts died faster than the uninfected mosquitoes. Mosquitoes carrying sporozoites had the same survival as the uninfected mosquitoes.

In 2012, Vezilier *et al.*[48] investigated the role of egg-laying on the mortality of malaria-infected mosquitoes. Using a naturally occurring mosquito-parasite pair, they found no difference in survival between infected and uninfected groups when mosquitoes were not allowed to lay eggs. However, when egg-laying was allowed, infected mosquitoes laid fewer eggs but lived longer than the uninfected controls. They surmised that the parasite redirects the mosquitoes’energy away from egg laying into prolonging life. This highlights the complex parasite-host interactions which may affect mosquito survival.

Excess mortality in heavy *P. knowlesi* infections might be due to a stress on the mosquito midgut from the large number of *P. knowlesi* oocysts. This stress may peak around day 7 when oocysts reach their full size. Stress from oocysts may combine with other stresses that were not measured, such as bacterial or fungal infection, or sub-optimal culture conditions.

### Infectivity of mosquitoes carrying *P. knowlesi*

Data on the high infectivity of *P. knowlesi*-carrying mosquitoes is consistent with the early observations of Chin *et al.*[36], who found that a bite from a single infected mosquito was able to infect the one human volunteer tested. In our hands, a single exposure to a mosquito carrying *P. knowlesi* sporozoites caused a malaria infection in 8/8 attempts. Most of these infections by single bite were from mosquitoes with only 11-100 sporozoites in their salivary glands. This indicates that the *P. knowlesi* sporozoites from lightly infected mosquitoes are quite virulent. The data show that 4/10 monkeys exposed to *P. knowlesi*-infected mosquitoes developed malaria in the absence of a detected blood meal. This means that measurements of exposures to *P. knowlesi* malaria should not be limited to counting mosquitoes with both blood meals and sporozoites, as this may underestimate the true sporozoite inoculum.

Chin *et al.*[36] noted a dose response effect in their five human subjects, with more infected bites leading to earlier detection of parasites in the blood. The data in 30 monkeys confirm this finding. Our interpretation is that fewer infective bites lead to fewer sporozoites reaching the liver and fewer liver-stage schizonts. This translates into fewer merozoites released from the liver, and a longer time until parasites are first detected in the blood. Anything that reduces the number of sporozoites reaching the liver should have the same effect. Therefore, delay to first parasitaemia is a valuable way to evaluate partial efficacy of vaccines directed at *P. knowlesi* pre-erythrocytic stages.

A *P. knowlesi* mosquito bite challenge was used here in a monkey vaccine trial. 17 monkeys, six control and 11 vaccinated animals, were exposed to five *P. knowlesi*-infected mosquitoes for 15 minutes. The vaccine did not protect any of the monkeys, and all the control and vaccinated monkeys developed malaria parasites in their blood 7 to 9 days after exposure. Although the preliminary data suggested that a single bite from a *P. knowlesi*-infected mosquito was adequate exposure to infect controls, it was decided to aim for at least two infected bites for each animal. Two bites were chosen instead of one, as with data from only eight animals there was not great confidence that a single bite would always transmit malaria. This study was also influenced by data from murine malaria, where a single bite from an infected mosquito only produced malaria infection in 39% of mice [29]. By estimating that 90% of the batch of mosquitoes was carrying *P. knowlesi* sporozoites, and estimating that 50% of mosquitoes would succeed in taking a blood meal, it was calculated (using binomial probabilities) that exposing each monkey to five mosquitoes would deliver at least two infective bites to each animal with probability of about 75%. In retrospect, exposing each monkey to fewer infective mosquitoes (perhaps 3 or 4) would have provided at least one infective bite which would have been an adequate challenge. With more experience in vaccine challenges by mosquito bite using *P. knowlesi,* the authors hope to generate the data to answer this question.

*Plasmodium knowlesi* sporozoites in rhesus seem to be more infectious than *Plasmodium falciparum* sporozoites in humans. While the data show that one infective bite is probably adequate to infect a monkey, human studies have shown that volunteers exposed to two bites of *P. falciparum*-infected mosquitoes do not always become infected [49,50]. This has led to the standard of five bites by *P. falciparum*-infected mosquitoes for laboratory-based malaria vaccine trials [51]. The reasons for this difference in virulence between *P. knowlesi* and *P. falciparum* are not understood. In the wild, most human infections by *P falciparum* and *P. knowlesi* are probably both caused by a single bite by a Plasmodium-carrying mosquito.

Adding methylparaben to the sugar water of infected mosquitoes did not improve survival. The original paper describing methylparaben [37] in mosquitoes carrying *Plasmodia* indicated that survival benefits were not evident when sugar pads were changed daily. As sugar pads were changed every day in our insectaries, the failure to see improvement was not surprising.

## Conclusions

Mosquitoes fed on donor monkeys with a high *P. knowlesi* parasitaemia have poor survival. Lower donor parasitaemias allow better mosquito survival and adequate sporozoite production. *Plasmodium knowlesi*-infected mosquitoes are extremely infectious, and a single bite is able to infect a monkey. Based on these results, a plan has been formulated for future *P. knowlesi* challenges of malaria vaccines by mosquito bite: (1) *Anopheles crascens* or *An. dirus* X mosquitoes will be used; (2) mosquitoes will be fed on a donor monkey when parasitaemia is from 0.2 to 2.0%; and (3) the goal of exposure will be one mosquito having both *P. knowlesi* sporozoites in the salivary glands of grade 2 (11-100) and ingested blood in the midgut. To achieve this single infectious bite, challenge will start with one exposure to mosquitoes, with a second exposure if necessary. For the first exposure, the number of infective *P. knowlesi* mosquitoes exposed to each monkey will be calculated from dissection of 10 mosquitoes to estimate the percentage having at least grade 2 (11-100) sporozoites in the salivary glands, and assuming a 50% bite rate. (For example, if 100% of mosquitoes are carrying *P. knowlesi* sporozoites, each monkey would be exposed to two mosquitoes.) All monkeys will receive this exposure for 15-30 minutes to allow as many mosquitoes to take a blood meal as possible. After exposure, all mosquitoes will be dissected to determine the number of mosquitoes carrying *P. knowlesi* sporozoites that ingested blood from each monkey and the actual bite rate will be calculated. If any animals have not received one infective bite in the first exposure, these monkeys will be exposed to *P. knowlesi*-infected mosquitoes from the same batch a second time. The second exposure may be on the same day as the first exposure or the day following, the timing depending on the ability of the monkeys to tolerate anesthesia. The number of mosquitoes used for the second exposure will be calculated using the rate of sporozoite infection and the actual bite rate from the first exposure.

## Abbreviations

MPB: Methylparaben; WRAIR/NMRC: Walter Reed Army Institute of Research/Naval Medical Research Center; LMIV: Laboratory of Malaria Immunology and Vaccinology; LMVR: Laboratory of Malaria and Vector Research.

## Competing interests

The authors declare that they have no competing interests.

## Authors’ contributions

JM, WW, DF, and LL fed mosquitoes on monkeys. JC, CS, MD, TS, OM, SO-G, KLZ and JH counted parasites in mosquitoes or on blood films. MF and GJ did the statistical analysis. RG, TR, EV, JR and PD provided advice and guidance. All authors read and approved the final manuscript.

## Supplementary Material

Additional file 1 **Experiments 1 to 5 of mosquito survival after *P. knowlesi *** **infection.** Description of data: This spreadsheet provides the mosquito species, donor monkey parasitaemia, total number of mosquitoes alive, and the oocyst and sporozoite dissection results for five separate experiments. Click here for file

Additional file 2 **Monkey infections after exposures to *P. knowlesi* infected mosquitoes.** Description of data: This spreadsheet provides the infection results of 49 monkey exposures to P. knowlesi infected mosquitoes, as well as the data from the mosquito dissections. Click here for file
